# Building evidence and capacity in global health palliative care

**DOI:** 10.3332/ecancer.2022.1378

**Published:** 2022-04-28

**Authors:** Richard Harding

**Affiliations:** Cicely Saunders Institute, Florence Nightingale Faculty of Nursing, Midwifery and Palliative Care, King’s College London, Bessemer Road, London SE5 9PJ, UK

**Keywords:** palliative care, global health, capacity, cancer

## Abstract

To achieve universal health coverage goals, access to quality palliative care must be rapidly scaled up in low and middle-income countries. By 2060, people living with cancer at the end of life will be the major contributors to serious health-related suffering. Major developments have occurred in the science of palliative care research in low- and middle-income countries (LMIC), from the development and validation of outcome measures to the delivery of randomised controlled trials. While the evidence has demonstrated context-specific needs among patients and families facing life-limiting illness, there are also many commonalities. Specific areas of leadership in the field have emerged from LMIC in HIV palliative care and in care for children. These innovations offer enormous potential for adaptation in high income countries. International partnerships in research, founded on mutually beneficial learning and capacity building, are central to achieving universal health coverage goals.

## The rising need for cancer palliative care in low- and middle-income countries

The universal health coverage goals include palliative care as an essential health service [1]. This is intended to address the global inequity in palliative care identified by the recent Lancet Commission and the WHO Atlas of palliative care, which found that 80% of those who would benefit from palliative care live in low- and middle-income countries (LMIC), while <10% receive it [2, 3]. Projections using the WHO mortality data suggest that by 2060, 83% of all deaths with serious health-related suffering will occur in LMIC (5.14 million in low-income countries, 16.84 million in lower-middle and 17.93 million in upper-middle) [4].The increase will be greatest in low-income countries (155%). Progressive illnesses cause highly prevalent and burdensome psychological, economic/social, physical and spiritual concerns to patients in low and middle-income countries, and to their largely female family caregivers [5–9], with financial implications for families and systems [10–13]. A review of palliative care needs in Africa identified context-specific needs [14]. These include managing pregnancy and breastfeeding; preventing infection transmission (physical); health literacy needs; worrying about medical bills (psychological); isolation and stigma; overwhelmed families needing a break; struggling to pay children’s school fees and selling assets (social and practical needs); and rites associated with cultural and religious beliefs (spiritual).

Despite unnecessary and growing suffering among patients and families facing progressive illness in LMIC, there is little evidence for palliative care interventions [15]. As populations age, cancer mortality also increases. 70% of incident cancer is in LMIC [16, 17]. Cancer will be the most common cause of death with serious health-related suffering by 2060 [18]. For low-income countries, this will be a 407% increase from 2016 deaths per year to 1.65 million people; and for lower-middle income countries, a 169% increase to 4.76 million. Cancer deaths in Africa are predicted to double between 2012 and 2030 to an estimated 889,604 [19, 20].

## Methodological developments

Over the past two decades a body of evidence has emerged from LMIC to guide research, policy and health system development. Importantly, in addition to the identification of needs and priorities among patients and their families living with life-limiting illness, effective interventions are emerging. In Kenya, an RCT of integrated palliative care into existing routine care for patients on antiretroviral therapy improved psychosocial problems, mental health and quality of life [21]. Early findings from a feasibility trial of early integrated palliative care for cancer patients in Ethiopia showed promising outcomes [22]. These advances enable the development in methods specific to the population and to the region, specifically in the area of ethical trials in LMIC [23]. A major advancement has been in the field of measurement, with an outcome measure specific to the African context being used to measure trial endpoints. The application of rigorous psychometric science to the development and testing of outcome measurement has led to widespread adoption in both clinical practice and research [24–27]. Additional validation of a hand-scoring system for the use of low literacy has improved access for patients to identify the burden of their symptoms and concerns [28].

## Mutual learning and contextual development

Measurement for paediatric palliative care has been identified as a global priority, yet little advancement has been made [29]. The world’s first patient-centred outcome measures for children and young people with life-limiting and life-threatening conditions has come from sub-Saharan Africa [30]. This major scientific advancement is based on a systematic review which found a number of potential global core outcomes of interest for children and young people [31]. This advancement from Africa is now being adopted in Europe, building on best available evidence for health measurement design for children and young people [32]. M-health for palliative care is another area of intervention being more rapidly developed in LMIC than in high-income countries [33], with engagement by health policymakers [34]. Evidence from pilot studies in India and Africa found that community health workers felt empowered and were more able to assess their clients when using a patient-centred outcome measure with an app, and palliative care services found that a dashboard of outcome data enabled them to better allocate limited staff resources to those in greatest need [35].

Interventions are also adaptable. Evidence from Africa on effective self-management of pain among people living with HIV [36] is now being translated to the UK setting [37]. Interventions must reflect cultural preferences and practices. For example, evidence shows that in India there is a preference for information to be disclosed to patients by family members, and so communication interventions should reflect that mechanism [38].

The stages of adaptation and designing for context are crucial. The evidence underpinning many core interventions in palliative care may be rooted in very Western contexts and conceptual models, for example, a logic model of the mechanisms and concepts underpinning ACP found an almost exclusively Western evidence base [39]. Measurement may also require adaptation to particular populations, for example, evidence of specific needs among refugees in the Middle East with advanced cancer informs of a specific adaptation of the Integrated Palliative Outcome Scale to reflect their self-reported compounded trauma [40].

## Conclusion

In conclusion, strong cross-national partnerships in research can lead to the development and implementation of locally relevant evidence, while also providing other regions with novel ideas for adaptation to context. International palliative care research must be founded on clearly articulated agreements for mutually beneficial and successful partnerships [41].

## Conflicts of interest

None to declare.

## Funding

RH is funded by the National Institute of Health Research (NIHR) Global Health Research Unit on Health System Strengthening in Sub-Saharan Africa, King’s College London (GHRU 16/136/54) using UK aid from the UK government to support global health research. RH is also funded by the UK Research and Innovation Global Challenges Research Fund: Research for Health in Conflict-Middle East and North Africa region (R4HC-MENA) (grant number ES/P010962/1). The views expressed in this publication are those of the author(s) and not necessarily those of the NIHR, the Department of Health and Social Care or UKRI.
